# Supplementary material for: Prediction and developing of shear strength of reinforced high strength concrete beams with and without steel fibers using multiple mathematical models
Source: PLoS One. 2022 Mar 31;17(3):e0265677. doi: 10.1371/journal.pone.0265677 (PMC8970403; doi:10.1371/journal.pone.0265677)
Supplement: S1 Table — (DOCX) [file pone.0265677.s001.docx]

| S1 Table. Experimental database for shear strength of HSC beams. | | | | | | |  |  |  |  |  |
| --- | --- | --- | --- | --- | --- | --- | --- | --- | --- | --- | --- |
|  |  |  |  |  |  |  |  |  |  | Exp. |  |
| No. | Reference | b (mm) | d (mm) | a (mm) | a/d | d/a | fc' (Mpa) | ρl | ρl *d/a | Vcr (KN) Exp | Vu (KN) |
| 1 | 9 | 100 | 180 | 510 | 2.833 | 0.353 | 21 | 0.0131 | 0.00462 | 36 | 42 |
| 2 |  | 100 | 180 | 510 | 2.833 | 0.353 | 26 | 0.0131 | 0.00462 | 40 | 46 |
| 3 |  | 100 | 180 | 510 | 2.833 | 0.353 | 27 | 0.0131 | 0.00462 | 38 | 46 |
| 4 |  | 100 | 180 | 510 | 2.833 | 0.353 | 29 | 0.0131 | 0.00462 | 56 | 58 |
| 5 |  | 100 | 180 | 510 | 2.833 | 0.353 | 67 | 0.0131 | 0.00462 | 76 | 82 |
| 6 |  | 100 | 180 | 510 | 2.833 | 0.353 | 74 | 0.0131 | 0.00462 | 72 | 79 |
| 7 |  | 100 | 180 | 510 | 2.833 | 0.353 | 71 | 0.0131 | 0.00462 | 62 | 76 |
| 8 |  | 100 | 180 | 510 | 2.833 | 0.353 | 72 | 0.0131 | 0.00462 | 56 | 64 |
| 9 | 51 | 50 | 180 | 350 | 4.4 | 0.227273 | 76.5 | 0.0133 | 0.00302 | 4.1 | 12.42 |
| 10 | 52 | 375 | 655 | 2150 | 3.28 | 0.304878 | 36 | 0.0288 | 0.00878 | 245 | 249 |
| 11 |  | 375 | 655 | 2150 | 3.28 | 0.304878 | 36 | 0.0288 | 0.00878 | 260 | 457 |
| 12 |  | 375 | 655 | 2150 | 3.28 | 0.304878 | 36 | 0.0288 | 0.00878 | 200 | 363 |
| 13 |  | 375 | 655 | 2150 | 3.28 | 0.304878 | 36 | 0.0288 | 0.00878 | 254 | 483 |
| 14 |  | 375 | 655 | 2150 | 3.28 | 0.304878 | 67 | 0.0288 | 0.00878 | 289 | 296 |
| 15 |  | 375 | 655 | 2150 | 3.28 | 0.304878 | 67 | 0.0288 | 0.00878 | 289 | 405 |
| 16 |  | 375 | 655 | 2150 | 3.28 | 0.304878 | 67 | 0.0288 | 0.00878 | 289 | 552 |
| 17 |  | 375 | 655 | 2150 | 3.28 | 0.304878 | 67 | 0.0288 | 0.00878 | 289 | 689 |
| 18 |  | 375 | 655 | 2150 | 3.28 | 0.304878 | 87 | 0.0288 | 0.00878 | 311 | 327 |
| 19 |  | 375 | 655 | 2150 | 3.28 | 0.304878 | 87 | 0.0288 | 0.00878 | 311 | 483 |
| 20 |  | 375 | 655 | 2150 | 3.28 | 0.304878 | 87 | 0.0288 | 0.00878 | 311 | 598 |
| 21 |  | 375 | 655 | 2150 | 3.28 | 0.304878 | 87 | 0.0288 | 0.00878 | 334 | 721 |
| 22 | 53 | 200 | 359 | 1080.59 | 3.01 | 0.332226 | 49.9 | 0.0224 | 0.00744 | 95 | 99.69 |
| 23 |  | 200 | 353 | 1080.18 | 3.06 | 0.326797 | 49.9 | 0.0228 | 0.00745 | 85 | 172.64 |
| 24 |  | 200 | 351 | 1081.08 | 3.08 | 0.324675 | 49.9 | 0.0229 | 0.00744 | 90 | 242.07 |
| 25 |  | 200 | 351 | 1081.08 | 3.08 | 0.324675 | 49.9 | 0.0299 | 0.00971 | 110 | 246.34 |
| 26 |  | 200 | 359 | 1080.59 | 3.01 | 0.332226 | 49.9 | 0.0224 | 0.00744 | 85 | 129.65 |
| 27 |  | 200 | 359 | 1080.59 | 3.01 | 0.332226 | 60.8 | 0.0224 | 0.00744 | 104 | 108.14 |
| 28 |  | 200 | 353 | 1080.18 | 3.06 | 0.326797 | 60.8 | 0.0228 | 0.00745 | 95 | 179.74 |
| 29 |  | 200 | 351 | 1081.08 | 3.08 | 0.324675 | 60.8 | 0.0229 | 0.00744 | 100 | 258.78 |
| 30 |  | 200 | 359 | 1080.59 | 3.01 | 0.332226 | 68.9 | 0.0224 | 0.00744 | 99 | 99.93 |
| 31 |  | 200 | 353 | 1080.18 | 3.06 | 0.326797 | 68.9 | 0.0228 | 0.00745 | 95 | 203.94 |
| 32 |  | 200 | 351 | 1081.08 | 3.08 | 0.324675 | 68.9 | 0.0229 | 0.00744 | 95 | 269.35 |
| 33 |  | 200 | 351 | 1081.08 | 3.08 | 0.324675 | 68.9 | 0.0299 | 0.00971 | 100 | 235.23 |
| 34 |  | 200 | 359 | 1080.59 | 3.01 | 0.332226 | 87 | 0.0224 | 0.00744 | 117.85 | 117.85 |
| 35 |  | 200 | 353 | 1080.18 | 3.06 | 0.326797 | 87 | 0.0228 | 0.00745 | 110 | 225.55 |
| 36 |  | 200 | 351 | 1081.08 | 3.08 | 0.324675 | 87 | 0.0229 | 0.00744 | 110 | 253.64 |
| 37 |  | 200 | 351 | 1081.08 | 3.08 | 0.324675 | 87 | 0.0299 | 0.00971 | 85 | 266.53 |
| 38 |  | 200 | 359 | 1080.59 | 3.01 | 0.332226 | 87 | 0.0224 | 0.00744 | 85 | 140.09 |
| 39 | 54 | 120 | 175 | 400 | 2.3 | 0.434783 | 30 | 0.0187 | 0.00813 | 33.5 | 64 |
| 40 |  | 120 | 175 | 400 | 2.3 | 0.434783 | 30 | 0.0187 | 0.00813 | 30 | 60 |
| 41 |  | 120 | 175 | 400 | 2.3 | 0.434783 | 30 | 0.0187 | 0.00813 | 27.5 | 56 |
| 42 |  | 120 | 175 | 400 | 2.3 | 0.434783 | 30 | 0.0187 | 0.00813 | 25 | 50 |
| 43 |  | 120 | 175 | 400 | 2.3 | 0.434783 | 30 | 0.0187 | 0.00813 | 24 | 45 |
| 44 |  | 120 | 175 | 400 | 2.3 | 0.434783 | 38 | 0.0187 | 0.00813 | 37.5 | 67.5 |
| 45 |  | 120 | 175 | 400 | 2.3 | 0.434783 | 38 | 0.0187 | 0.00813 | 35 | 65 |
| 46 |  | 120 | 175 | 400 | 2.3 | 0.434783 | 38 | 0.0187 | 0.00813 | 33.5 | 61 |
| 47 |  | 120 | 175 | 400 | 2.3 | 0.434783 | 38 | 0.0187 | 0.00813 | 32.5 | 55 |
| 48 |  | 120 | 175 | 400 | 2.3 | 0.434783 | 38 | 0.0187 | 0.00813 | 30 | 50 |
| 49 |  | 120 | 175 | 400 | 2.3 | 0.434783 | 51 | 0.0187 | 0.00813 | 35 | 76.5 |
| 50 |  | 120 | 175 | 400 | 2.3 | 0.434783 | 51 | 0.0187 | 0.00813 | 32.5 | 71.5 |
| 51 |  | 120 | 175 | 400 | 2.3 | 0.434783 | 51 | 0.0187 | 0.00813 | 30 | 65 |
| 52 |  | 120 | 175 | 400 | 2.3 | 0.434783 | 51 | 0.0187 | 0.00813 | 30 | 61 |
| 53 |  | 120 | 175 | 400 | 2.3 | 0.434783 | 51 | 0.0187 | 0.00813 | 28 | 55.5 |
| 54 |  | 120 | 175 | 400 | 2.3 | 0.434783 | 64 | 0.0187 | 0.00813 | 77.5 | 77.5 |
| 55 |  | 120 | 175 | 400 | 2.3 | 0.434783 | 64 | 0.0187 | 0.00813 | 72.5 | 72.5 |
| 56 |  | 120 | 175 | 400 | 2.3 | 0.434783 | 64 | 0.0187 | 0.00813 | 66 | 66 |
| 57 |  | 120 | 175 | 400 | 2.3 | 0.434783 | 64 | 0.0187 | 0.00813 | 62.5 | 62.5 |
| 58 |  | 120 | 175 | 400 | 2.3 | 0.434783 | 64 | 0.0187 | 0.00813 | 57.5 | 57.5 |
| 59 |  | 120 | 175 | 400 | 2.3 | 0.434783 | 76.5 | 0.0187 | 0.00813 | 75 | 75 |
| 60 |  | 120 | 175 | 400 | 2.3 | 0.434783 | 76.5 | 0.0187 | 0.00813 | 71 | 71 |
| 61 |  | 120 | 175 | 400 | 2.3 | 0.434783 | 76.5 | 0.0187 | 0.00813 | 65 | 65 |
| 62 |  | 120 | 175 | 400 | 2.3 | 0.434783 | 76.5 | 0.0187 | 0.00813 | 61.5 | 61.5 |
| 63 |  | 120 | 175 | 400 | 2.3 | 0.434783 | 76.5 | 0.0187 | 0.00813 | 55.5 | 55.5 |
| 64 | 2,14 | 120 | 180 | 480.6 | 2.67 | 0.374532 | 35 | 0.017 | 0.00637 | 22 | 54 |
| 65 |  | 120 | 180 | 480.6 | 2.67 | 0.374532 | 35 | 0.024 | 0.00899 | 27 | 50 |
| 66 |  | 120 | 180 | 480.6 | 2.67 | 0.374532 | 35 | 0.032 | 0.01199 | 32 | 71.5 |
| 67 |  | 120 | 240 | 480 | 2 | 0.5 | 35 | 0.017 | 0.0085 | 30 | 61.5 |
| 68 |  | 120 | 240 | 480 | 2 | 0.5 | 35 | 0.024 | 0.012 | 36 | 89.5 |
| 69 |  | 120 | 240 | 480 | 2 | 0.5 | 35 | 0.032 | 0.016 | 40 | 115 |
| 70 |  | 120 | 300 | 450 | 1.5 | 0.666667 | 35 | 0.018 | 0.012 | 50 | 110 |
| 71 |  | 120 | 300 | 450 | 1.5 | 0.666667 | 35 | 0.024 | 0.016 | 52.5 | 175 |
| 72 |  | 120 | 300 | 450 | 1.5 | 0.666667 | 35 | 0.032 | 0.02133 | 52.5 | 192.5 |
| 73 |  | 120 | 360 | 450 | 1.25 | 0.8 | 35 | 0.015 | 0.012 | 50 | 135 |
| 74 |  | 120 | 360 | 450 | 1.25 | 0.8 | 35 | 0.025 | 0.02 | 70 | 200 |
| 75 |  | 120 | 360 | 450 | 1.25 | 0.8 | 35 | 0.033 | 0.0264 | 80 | 220 |
| 76 |  | 120 | 360 | 478.8 | 1.33 | 0.75188 | 35 | 0.032 | 0.02406 | 70 | 225 |
| 77 |  | 120 | 180 | 480.6 | 2.67 | 0.374532 | 90 | 0.017 | 0.00637 | 34.5 | 57.5 |
| 78 |  | 120 | 180 | 480.6 | 2.67 | 0.374532 | 90 | 0.024 | 0.00899 | 32 | 60 |
| 79 |  | 120 | 180 | 480.6 | 2.67 | 0.374532 | 90 | 0.032 | 0.01199 | 40 | 74 |
| 80 |  | 120 | 240 | 480 | 2 | 0.5 | 90 | 0.017 | 0.0085 | 40 | 83.5 |
| 81 |  | 120 | 240 | 480 | 2 | 0.5 | 90 | 0.024 | 0.012 | 45 | 108.5 |
| 82 |  | 120 | 240 | 480 | 2 | 0.5 | 90 | 0.032 | 0.016 | 55 | 152.5 |
| 83 |  | 120 | 300 | 450 | 1.5 | 0.666667 | 90 | 0.018 | 0.012 | 60 | 160 |
| 84 |  | 120 | 300 | 450 | 1.5 | 0.666667 | 90 | 0.024 | 0.016 | 67.5 | 210 |
| 85 |  | 120 | 300 | 450 | 1.5 | 0.666667 | 90 | 0.032 | 0.02133 | 70 | 192 |
| 86 |  | 120 | 360 | 450 | 1.25 | 0.8 | 90 | 0.015 | 0.012 | 70 | 205 |
| 87 |  | 120 | 360 | 450 | 1.25 | 0.8 | 90 | 0.025 | 0.02 | 100 | 315 |
| 88 |  | 120 | 360 | 450 | 1.25 | 0.8 | 90 | 0.033 | 0.0264 | 100 | 337.5 |
| 89 |  | 120 | 360 | 478.8 | 1.33 | 0.75188 | 90 | 0.032 | 0.02406 | 112.5 | 335 |
| 90 | 16 | 120 | 275 | 275 | 1 | 1 | 30 | 0.0103 | 0.0103 | 40.26 | 131.9 |
| 91 |  | 120 | 275 | 550 | 2 | 0.5 | 30 | 0.0103 | 0.00515 | 28.38 | 80.45 |
| 92 |  | 120 | 275 | 825 | 3 | 0.333333 | 30 | 0.0103 | 0.00343 | 23.43 | 67.22 |
| 93 |  | 120 | 275 | 1100 | 4 | 0.25 | 30 | 0.0103 | 0.00258 | 12.87 | 37.2 |
| 94 |  | 120 | 275 | 550 | 2 | 0.5 | 30 | 0.0103 | 0.00515 | 38.28 | 89.13 |
| 95 |  | 120 | 275 | 550 | 2 | 0.5 | 30 | 0.0103 | 0.00515 | 32.34 | 87.6 |
| 96 |  | 120 | 275 | 550 | 2 | 0.5 | 30 | 0.0103 | 0.00515 | 35.64 | 86.53 |
| 97 |  | 120 | 275 | 550 | 2 | 0.5 | 30 | 0.0103 | 0.00515 | 30.69 | 82.5 |
| 98 |  | 120 | 275 | 550 | 2 | 0.5 | 50 | 0.0103 | 0.00515 | 46.53 | 111.21 |
| 99 |  | 120 | 275 | 550 | 2 | 0.5 | 50 | 0.0103 | 0.00515 | 39.6 | 105.4 |
| 100 |  | 120 | 275 | 550 | 2 | 0.5 | 50 | 0.0103 | 0.00515 | 33 | 98.27 |
| 101 |  | 120 | 275 | 275 | 1 | 1 | 80 | 0.0103 | 0.0103 | 61.05 | 277.04 |
| 102 |  | 120 | 275 | 550 | 2 | 0.5 | 80 | 0.0103 | 0.00515 | 35.64 | 114.08 |
| 103 |  | 120 | 275 | 825 | 3 | 0.333333 | 80 | 0.0103 | 0.00343 | 25.41 | 78.34 |
| 104 |  | 120 | 275 | 1100 | 4 | 0.25 | 80 | 0.0103 | 0.00258 | 13.86 | 48.38 |
| 105 |  | 120 | 275 | 550 | 2 | 0.5 | 80 | 0.0103 | 0.00515 | 50.82 | 123.222 |
| 106 |  | 120 | 275 | 550 | 2 | 0.5 | 80 | 0.0103 | 0.00515 | 45.87 | 119.658 |
| 107 |  | 120 | 275 | 550 | 2 | 0.5 | 80 | 0.0103 | 0.00515 | 41.91 | 120.68 |
| 108 |  | 120 | 275 | 550 | 2 | 0.5 | 80 | 0.0103 | 0.00515 | 38.61 | 119.163 |
| 109 | 17 | 150 | 298.5 | 895.5 | 3 | 0.333333 | 22.08 | 0.0329 | 0.01097 | 44.775 | 75.04 |
| 110 |  | 150 | 298.5 | 895.5 | 3 | 0.333333 | 39.83 | 0.0329 | 0.01097 | 61.45 | 92.33 |
| 111 |  | 150 | 298.5 | 895.5 | 3 | 0.333333 | 59.73 | 0.0329 | 0.01097 | 70.1 | 96.34 |
| 112 |  | 150 | 298.5 | 895.5 | 3 | 0.333333 | 82.97 | 0.0329 | 0.01097 | 78.74 | 109.62 |
| 113 |  | 150 | 298.5 | 895.5 | 3 | 0.333333 | 27.925 | 0.0329 | 0.01097 | 56.82 | 93.87 |
| 114 |  | 150 | 298.5 | 895.5 | 3 | 0.333333 | 47.09 | 0.0329 | 0.01097 | 78.74 | 118.58 |
| 115 |  | 150 | 298.5 | 895.5 | 3 | 0.333333 | 68.6 | 0.0329 | 0.01097 | 78.74 | 148.84 |
| 116 |  | 150 | 298.5 | 895.5 | 3 | 0.333333 | 81.92 | 0.0329 | 0.01097 | 78.74 | 113.945 |
| 117 |  | 150 | 298.5 | 895.5 | 3 | 0.333333 | 28.71 | 0.0329 | 0.01097 | 65.773 | 136.8 |
| 118 |  | 150 | 298.5 | 895.5 | 3 | 0.333333 | 46.57 | 0.0329 | 0.01097 | 70.1 | 131.237 |
| 119 |  | 150 | 298.5 | 895.5 | 3 | 0.333333 | 69.56 | 0.0329 | 0.01097 | 78.74 | 159.03 |
| 120 |  | 150 | 298.5 | 895.5 | 3 | 0.333333 | 82.77 | 0.0329 | 0.01097 | 78.74 | 147.6 |
| 121 | 18 | 150 | 250 | 750 | 3 | 0.333333 | 37.8 | 0.0153 | 0.0051 | 43.125 |  |
| 122 |  | 150 | 250 | 750 | 3 | 0.333333 | 36.4 | 0.0153 | 0.0051 | 55.5 |  |
| 123 |  | 150 | 250 | 750 | 3 | 0.333333 | 36.4 | 0.0153 | 0.0051 | 56.25 |  |
| 124 |  | 150 | 500 | 1500 | 3 | 0.333333 | 37.4 | 0.0153 | 0.0051 | 116.25 |  |
| 125 |  | 150 | 500 | 1500 | 3 | 0.333333 | 36.4 | 0.0153 | 0.0051 | 78.75 |  |
| 126 |  | 150 | 500 | 1500 | 3 | 0.333333 | 36.4 | 0.0153 | 0.0051 | 87 |  |
| 127 |  | 350 | 1000 | 3000 | 3 | 0.333333 | 35.7 | 0.0136 | 0.00453 | 318.5 |  |
| 128 |  | 350 | 1000 | 3000 | 3 | 0.333333 | 34.7 | 0.0136 | 0.00453 | 297.5 |  |
| 129 |  | 350 | 1000 | 3000 | 3 | 0.333333 | 34.7 | 0.0136 | 0.00453 | 332.5 |  |
| 130 |  | 150 | 250 | 750 | 3 | 0.333333 | 68.8 | 0.0153 | 0.0051 | 51.375 |  |
| 131 |  | 150 | 250 | 750 | 3 | 0.333333 | 51.9 | 0.0153 | 0.0051 | 65.25 |  |
| 132 |  | 150 | 250 | 750 | 3 | 0.333333 | 51.9 | 0.0153 | 0.0051 | 53.625 |  |
| 133 |  | 150 | 500 | 1500 | 3 | 0.333333 | 69.4 | 0.0153 | 0.0051 | 87 |  |
| 134 |  | 150 | 500 | 1500 | 3 | 0.333333 | 51.9 | 0.0153 | 0.0051 | 108 |  |
| 135 |  | 150 | 500 | 1500 | 3 | 0.333333 | 51.9 | 0.0153 | 0.0051 | 113.25 |  |
| 136 |  | 350 | 1000 | 3000 | 3 | 0.333333 | 59.2 | 0.0136 | 0.00453 | 315 |  |
| 137 |  | 350 | 1000 | 3000 | 3 | 0.333333 | 53.3 | 0.0136 | 0.00453 | 339.5 |  |
| 138 |  | 350 | 1000 | 3000 | 3 | 0.333333 | 53 | 0.0136 | 0.00453 | 339.5 |  |
| 139 |  | 150 | 250 | 750 | 3 | 0.333333 | 101 | 0.0153 | 0.0051 | 40.125 |  |
| 140 |  | 150 | 250 | 750 | 3 | 0.333333 | 92.9 | 0.0153 | 0.0051 | 56.25 |  |
| 141 |  | 150 | 250 | 750 | 3 | 0.333333 | 92.9 | 0.0153 | 0.0051 | 46.875 |  |
| 142 |  | 150 | 500 | 1500 | 3 | 0.333333 | 102 | 0.0153 | 0.0051 | 86.25 |  |
| 143 |  | 150 | 500 | 1500 | 3 | 0.333333 | 92.9 | 0.0153 | 0.0051 | 82.5 |  |
| 144 |  | 150 | 500 | 1500 | 3 | 0.333333 | 92.9 | 0.0153 | 0.0051 | 97.5 |  |
| 145 |  | 350 | 1000 | 3000 | 3 | 0.333333 | 103 | 0.0136 | 0.00453 | 280 |  |
| 146 |  | 350 | 1000 | 3000 | 3 | 0.333333 | 89.9 | 0.0136 | 0.00453 | 262.5 |  |
| 147 |  | 350 | 1000 | 3000 | 3 | 0.333333 | 92.1 | 0.0136 | 0.00453 | 280 |  |
| 148 |  | 150 | 500 | 1000 | 2 | 0.5 | 37.2 | 0.0153 | 0.00765 | 99.75 |  |
| 149 |  | 150 | 500 | 1000 | 2 | 0.5 | 36.4 | 0.0153 | 0.00765 | 97.5 |  |
| 150 |  | 150 | 500 | 1000 | 2 | 0.5 | 36.4 | 0.0153 | 0.00765 | 104.25 |  |
| 151 |  | 150 | 500 | 2000 | 4 | 0.25 | 37.7 | 0.0153 | 0.00383 | 70.5 |  |
| 152 |  | 150 | 500 | 2000 | 4 | 0.25 | 36.4 | 0.0153 | 0.00383 | 84.75 |  |
| 153 |  | 150 | 500 | 2000 | 4 | 0.25 | 36.4 | 0.0153 | 0.00383 | 88.5 |  |
| 154 |  | 150 | 500 | 1000 | 2 | 0.5 | 69.3 | 0.0153 | 0.00765 | 98.25 |  |
| 155 |  | 150 | 500 | 1000 | 2 | 0.5 | 51.9 | 0.0153 | 0.00765 | 122.25 |  |
| 156 |  | 150 | 500 | 1000 | 2 | 0.5 | 51.9 | 0.0153 | 0.00765 | 114.75 |  |
| 157 |  | 150 | 500 | 2000 | 4 | 0.25 | 69.8 | 0.0153 | 0.00383 | 81.75 |  |
| 158 |  | 150 | 500 | 2000 | 4 | 0.25 | 51.9 | 0.0153 | 0.00383 | 90.75 |  |
| 159 |  | 150 | 500 | 1000 | 2 | 0.5 | 102 | 0.0153 | 0.00765 | 105.75 |  |
| 160 |  | 150 | 500 | 1000 | 2 | 0.5 | 92.9 | 0.0153 | 0.00765 | 94.5 |  |
| 161 |  | 150 | 500 | 1000 | 2 | 0.5 | 92.9 | 0.0153 | 0.00765 | 103.5 |  |
| 162 |  | 150 | 500 | 2000 | 4 | 0.25 | 101 | 0.0153 | 0.00383 | 72.75 |  |
| 163 |  | 150 | 500 | 2000 | 4 | 0.25 | 92.9 | 0.0153 | 0.00383 | 78.75 |  |
| 164 |  | 150 | 500 | 2000 | 4 | 0.25 | 92.9 | 0.0153 | 0.00383 | 84 |  |
| 165 | 19 | 125 | 100 | 300 | 3 | 0.333333 | 65 | 0.008 | 0.00267 | 8 | 30 |
| 166 |  | 125 | 100 | 300 | 3 | 0.333333 | 65 | 0.008 | 0.00267 | 6 | 18 |
| 167 |  | 125 | 100 | 500 | 5 | 0.2 | 65 | 0.008 | 0.0016 | 6 | 34 |
| 168 |  | 125 | 100 | 500 | 5 | 0.2 | 65 | 0.008 | 0.0016 | 8 | 16 |
| 169 |  | 125 | 100 | 300 | 3 | 0.333333 | 65 | 0.018 | 0.006 | 8 | 44 |
| 170 |  | 125 | 100 | 300 | 3 | 0.333333 | 65 | 0.018 | 0.006 | 6 | 24 |
| 171 |  | 125 | 100 | 500 | 5 | 0.2 | 65 | 0.018 | 0.0036 | 6 | 48 |
| 172 |  | 125 | 100 | 500 | 5 | 0.2 | 65 | 0.018 | 0.0036 | 6 | 18 |
| 173 |  | 125 | 100 | 300 | 3 | 0.333333 | 65 | 0.032 | 0.01067 | 10 | 58 |
| 174 |  | 125 | 100 | 300 | 3 | 0.333333 | 65 | 0.032 | 0.01067 | 6 | 26 |
| 175 |  | 125 | 100 | 500 | 5 | 0.2 | 65 | 0.032 | 0.0064 | 16 | 64 |
| 176 |  | 125 | 100 | 500 | 5 | 0.2 | 65 | 0.032 | 0.0064 | 8 | 24 |
| 177 | 55 | 305 | 539 | 1670 | 3.1 | 0.322581 | 36.414 | 0.0241 | 0.00777 | 178 | 339 |
| 178 |  | 305 | 539 | 1670 | 3.1 | 0.322581 | 36.414 | 0.0241 | 0.00777 | 178 | 222.217 |
| 179 |  | 305 | 539 | 1670 | 3.1 | 0.322581 | 72.345 | 0.0241 | 0.00777 | 222.217 | 263.03 |
| 180 |  | 305 | 539 | 1670 | 3.1 | 0.322581 | 72.345 | 0.0241 | 0.00777 | 214.28 | 316.319 |
| 181 |  | 305 | 539 | 1670 | 3.1 | 0.322581 | 55.862 | 0.0241 | 0.00777 | 178 | 383.21 |
| 182 |  | 305 | 539 | 1670 | 3.1 | 0.322581 | 55.862 | 0.0241 | 0.00777 | 191.605 | 191.605 |
| 183 |  | 305 | 539 | 1670 | 3.1 | 0.322581 | 51.31 | 0.0241 | 0.00777 | 178 | 281.172 |
| 184 |  | 305 | 539 | 1670 | 3.1 | 0.322581 | 51.31 | 0.0241 | 0.00777 | 196.14 | 258.5 |
|  |  |  |  |  |  |  |  |  |  |  |  |
| SUM |  |  |  |  |  |  |  |  |  |  |  |
