# Supplementary material for: Prediction and developing of shear strength of reinforced high strength concrete beams with and without steel fibers using multiple mathematical models
Source: PLoS One. 2022 Mar 31;17(3):e0265677. doi: 10.1371/journal.pone.0265677 (PMC8970403; doi:10.1371/journal.pone.0265677)
Supplement: S2 Table — (DOCX) [file pone.0265677.s002.docx]

| S2 Table. Experimental database for shear strength of HSC beams with steel fiber. | | | | | | | | | |  |  |  |  |  |  |  |
| --- | --- | --- | --- | --- | --- | --- | --- | --- | --- | --- | --- | --- | --- | --- | --- | --- |
|  |  |  |  |  |  |  |  |  |  |  |  |  |  |  |  |  |
|  |  |  |  |  |  |  |  |  |  |  |  |  |  |  | EXP. |  |
| No. | Reference | Vf% | (L/D)f | df | F | 1+F | b (mm) | d (mm) | a (mm) | a/d | d/a | fc' (MPa) | ρl | ρl * d/a | Vcr (KN) | Vu (KN) |
| 1 | 10 | 0 | 0 | 0 | 0 | 1 | 100 | 136 | 300 | 2.2 | 0.454545 | 65 | 0.0116 | 0.00527 | 30.31 | 42.565 |
| 2 |  | 0 | 0 | 0 | 0 | 1 | 100 | 136 | 300 | 2.2 | 0.454545 | 65 | 0.0116 | 0.00527 | 18.905 | 29.295 |
| 3 |  | 0.5 | 65 | 1 | 0.325 | 1.325 | 100 | 136 | 300 | 2.2 | 0.454545 | 64.2 | 0.0116 | 0.00527 | 20.715 | 43.105 |
| 4 |  | 1 | 65 | 1 | 0.65 | 1.65 | 100 | 136 | 300 | 2.2 | 0.454545 | 64.2 | 0.0116 | 0.00527 | 30.665 | 45.125 |
| 5 |  | 1 | 80 | 1 | 0.8 | 1.8 | 100 | 136 | 300 | 2.2 | 0.454545 | 60 | 0.0116 | 0.00527 | 30.65 | 48.875 |
| 6 |  | 2 | 65 | 1 | 1.3 | 2.3 | 100 | 136 | 300 | 2.2 | 0.454545 | 63.1 | 0.0116 | 0.00527 | 30.335 | 46.435 |
| 7 |  | 2 | 80 | 1 | 1.6 | 2.6 | 100 | 136 | 300 | 2.2 | 0.454545 | 65 | 0.0116 | 0.00527 | 35.395 | 52.5 |
| 8 |  | 3 | 65 | 1 | 1.95 | 2.95 | 100 | 136 | 300 | 2.2 | 0.454545 | 62.2 | 0.0116 | 0.00527 | 33.74 | 55.16 |
| 9 | 56 | 0 | 0 | 0 | 0 | 1 | 86.39 | 51 | 241 | 4.725 | 0.21164 | 120 | 0.016 | 0.00339 | 11.525 | 11.525 |
| 10 |  | 0 | 0 | 0 | 0 | 1 | 87.36 | 51 | 241 | 4.725 | 0.21164 | 120 | 0.032 | 0.00677 | 16.945 | 16.945 |
| 11 |  | 1 | 63.5 | 1 | 0.635 | 1.635 | 87.66 | 51 | 241 | 4.725 | 0.21164 | 150 | 0.016 | 0.00339 | 16.53 | 16.53 |
| 12 |  | 1 | 63.5 | 1 | 0.635 | 1.635 | 87.66 | 51 | 241 | 4.725 | 0.21164 | 150 | 0.032 | 0.00677 | 28.7 | 28.7 |
| 13 |  | 2 | 63.5 | 1 | 1.27 | 2.27 | 87.24 | 51 | 241 | 4.725 | 0.21164 | 145 | 0.016 | 0.00339 | 22.805 | 22.805 |
| 14 |  | 2 | 63.5 | 1 | 1.27 | 2.27 | 89.05 | 51 | 241 | 4.725 | 0.21164 | 145 | 0.032 | 0.00677 | 29.375 | 29.375 |
| 15 | 57 | 0 | 0 | 0 | 0 | 1 | 50 | 200 | 700 | 3.5 | 0.285714 | 121 | 0.008 | 0.00229 | 7.715 | 22.845 |
| 16 |  | 0 | 0 | 0 | 0 | 1 | 50 | 200 | 700 | 3.5 | 0.285714 | 121 | 0.012 | 0.00343 | 7.81 | 21.56 |
| 17 |  | 0 | 0 | 0 | 0 | 1 | 50 | 200 | 700 | 3.5 | 0.285714 | 121 | 0.017 | 0.00486 | 4.98 | 25.3 |
| 18 |  | 0 | 0 | 0 | 0 | 1 | 50 | 200 | 700 | 3.5 | 0.285714 | 121 | 0.022 | 0.00629 | 5.14 | 33.405 |
| 19 |  | 2 | 54.55 | 1 | 1.091 | 2.091 | 50 | 200 | 700 | 3.5 | 0.285714 | 143 | 0.008 | 0.00229 | 12.385 | 36.76 |
| 20 |  | 2 | 54.55 | 1 | 1.091 | 2.091 | 50 | 200 | 700 | 3.5 | 0.285714 | 143 | 0.012 | 0.00343 | 9.89 | 49.89 |
| 21 |  | 2 | 54.55 | 1 | 1.091 | 2.091 | 50 | 200 | 700 | 3.5 | 0.285714 | 143 | 0.017 | 0.00486 | 8.015 | 62.655 |
| 22 |  | 2 | 54.55 | 1 | 1.091 | 2.091 | 50 | 200 | 700 | 3.5 | 0.285714 | 143 | 0.022 | 0.00629 | 6.69 | 60.09 |
| 23 | 6 | 0 | 0 | 0 | 0 | 1 | 100 | 130 | 130 | 1 | 1 | 70 | 0.0725 | 0.0725 | 111.5 | 129 |
| 24 |  | 0 | 0 | 0 | 0 | 1 | 100 | 130 | 260 | 2 | 0.5 | 70 | 0.0725 | 0.03625 | 55 | 78.5 |
| 25 |  | 0 | 0 | 0 | 0 | 1 | 100 | 130 | 390 | 3 | 0.333333 | 70 | 0.0725 | 0.02417 | 30.5 | 60 |
| 26 |  | 0 | 0 | 0 | 0 | 1 | 100 | 130 | 520 | 4 | 0.25 | 70 | 0.0725 | 0.01813 | 25 | 55.5 |
| 27 |  | 0.4 | 75 | 0.5 | 0.15 | 1.15 | 100 | 130 | 130 | 1 | 1 | 70 | 0.0725 | 0.0725 | 115 | 146.5 |
| 28 |  | 0.4 | 75 | 0.5 | 0.15 | 1.15 | 100 | 130 | 260 | 2 | 0.5 | 70 | 0.0725 | 0.03625 | 69.5 | 90.5 |
| 29 |  | 0.4 | 75 | 0.5 | 0.15 | 1.15 | 100 | 130 | 390 | 3 | 0.333333 | 70 | 0.0725 | 0.02417 | 42.5 | 65.5 |
| 30 |  | 0.4 | 75 | 0.5 | 0.15 | 1.15 | 100 | 130 | 520 | 4 | 0.25 | 70 | 0.0725 | 0.01813 | 32.5 | 60 |
| 31 |  | 0.8 | 75 | 0.5 | 0.3 | 1.3 | 100 | 130 | 130 | 1 | 1 | 70 | 0.0725 | 0.0725 | 121 | 170 |
| 32 |  | 0.8 | 75 | 0.5 | 0.3 | 1.3 | 100 | 130 | 260 | 2 | 0.5 | 70 | 0.0725 | 0.03625 | 73 | 105 |
| 33 |  | 0.8 | 75 | 0.5 | 0.3 | 1.3 | 100 | 130 | 390 | 3 | 0.333333 | 70 | 0.0725 | 0.02417 | 54.5 | 75 |
| 34 |  | 0.8 | 75 | 0.5 | 0.3 | 1.3 | 100 | 130 | 520 | 4 | 0.25 | 70 | 0.0725 | 0.01813 | 45 | 62.5 |
| 35 |  | 1.2 | 75 | 0.5 | 0.45 | 1.45 | 100 | 130 | 130 | 1 | 1 | 70 | 0.0725 | 0.0725 | 125 | 177.5 |
| 36 |  | 1.2 | 75 | 0.5 | 0.45 | 1.45 | 100 | 130 | 260 | 2 | 0.5 | 70 | 0.0725 | 0.03625 | 77.5 | 112.5 |
| 37 |  | 1.2 | 75 | 0.5 | 0.45 | 1.45 | 100 | 130 | 390 | 3 | 0.333333 | 70 | 0.0725 | 0.02417 | 57.5 | 80 |
| 38 |  | 1.2 | 75 | 0.5 | 0.45 | 1.45 | 100 | 130 | 520 | 4 | 0.25 | 70 | 0.0725 | 0.01813 | 50 | 67.5 |
| 39 | 15 | 0 | 0 | 0 | 0 | 1 | 300 | 442 | 1350 | 3.054299 | 0.327407 | 67 | 0.0296 | 0.00969 | 51.4 | 226 |
| 40 |  | 0 | 0 | 0 | 0 | 1 | 300 | 442 | 1350 | 3.054299 | 0.327407 | 67 | 0.0296 | 0.00969 | 30.4 | 537.8 |
| 41 |  | 0 | 0 | 0 | 0 | 1 | 300 | 442 | 1350 | 3.054299 | 0.327407 | 67 | 0.0296 | 0.00969 | 35.8 | 493.7 |
| 42 |  | 0 | 0 | 0 | 0 | 1 | 300 | 442 | 1350 | 3.054299 | 0.327407 | 67 | 0.0296 | 0.00969 | 45.9 | 389.4 |
| 43 |  | 0.75 | 65 | 1 | 0.4875 | 1.4875 | 300 | 442 | 1350 | 3.054299 | 0.327407 | 72 | 0.0296 | 0.00969 | 56.2 | 411.1 |
| 44 | 23 | 1 | 75 | 1 | 0.75 | 1.75 | 125 | 215 | 430 | 2 | 0.5 | 92 | 0.0037 | 0.00187 | 45.15 |  |
| 45 |  | 1 | 75 | 1 | 0.75 | 1.75 | 125 | 215 | 860 | 4 | 0.25 | 92.6 | 0.0037 | 0.00094 | 24 |  |
| 46 |  | 1 | 75 | 1 | 0.75 | 1.75 | 125 | 215 | 1290 | 6 | 0.166667 | 93.7 | 0.0037 | 0.00062 | 15.05 |  |
| 47 |  | 0.5 | 75 | 1 | 0.375 | 1.375 | 125 | 215 | 215 | 1 | 1 | 99 | 0.0284 | 0.02835 | 244.3 |  |
| 48 |  | 0.5 | 75 | 1 | 0.375 | 1.375 | 125 | 215 | 430 | 2 | 0.5 | 99.1 | 0.0284 | 0.01418 | 129.54 |  |
| 49 |  | 0.5 | 75 | 1 | 0.375 | 1.375 | 125 | 215 | 860 | 4 | 0.25 | 95.4 | 0.0284 | 0.00709 | 61 |  |
| 50 |  | 0.5 | 75 | 1 | 0.375 | 1.375 | 125 | 215 | 1290 | 6 | 0.166667 | 95.83 | 0.0284 | 0.00473 | 52.4 |  |
| 51 |  | 1 | 75 | 1 | 0.75 | 1.75 | 125 | 215 | 215 | 1 | 1 | 95.3 | 0.0284 | 0.02835 | 342.4 |  |
| 52 |  | 1 | 75 | 1 | 0.75 | 1.75 | 125 | 215 | 430 | 2 | 0.5 | 95.3 | 0.0284 | 0.01418 | 162.86 |  |
| 53 |  | 1 | 75 | 1 | 0.75 | 1.75 | 125 | 215 | 860 | 4 | 0.25 | 97.53 | 0.0284 | 0.00709 | 85.2 |  |
| 54 |  | 1 | 75 | 1 | 0.75 | 1.75 | 125 | 215 | 1290 | 6 | 0.166667 | 100.5 | 0.0284 | 0.00473 | 52.7 |  |
| 55 |  | 1.5 | 75 | 1 | 1.125 | 2.125 | 125 | 215 | 215 | 1 | 1 | 96.4 | 0.0284 | 0.02835 | 374.91 |  |
| 56 |  | 1.5 | 75 | 1 | 1.125 | 2.125 | 125 | 215 | 430 | 2 | 0.5 | 96.6 | 0.0284 | 0.01418 | 193.77 |  |
| 57 |  | 1.5 | 75 | 1 | 1.125 | 2.125 | 125 | 215 | 860 | 4 | 0.25 | 97.1 | 0.0284 | 0.00709 | 94.33 |  |
| 58 |  | 1.5 | 75 | 1 | 1.125 | 2.125 | 125 | 215 | 1290 | 6 | 0.166667 | 101.32 | 0.0284 | 0.00473 | 53.2 |  |
| 59 |  | 1 | 75 | 1 | 0.75 | 1.75 | 125 | 215 | 430 | 2 | 0.5 | 94.5 | 0.0458 | 0.0229 | 180.87 |  |
| 60 |  | 1 | 75 | 1 | 0.75 | 1.75 | 125 | 215 | 860 | 4 | 0.25 | 93.8 | 0.0458 | 0.01145 | 104.275 |  |
| 61 |  | 1 | 75 | 1 | 0.75 | 1.75 | 125 | 215 | 1290 | 6 | 0.166667 | 95 | 0.0458 | 0.00763 | 78.74 |  |
|  |  |  |  |  |  |  |  |  |  |  |  |  |  |  |  |  |
| SUM |  |  |  |  |  |  |  |  |  |  |  |  |  |  |  |  |
