# Supplementary material for: Prediction and developing of shear strength of reinforced high strength concrete beams with and without steel fibers using multiple mathematical models
Source: PLoS One. 2022 Mar 31;17(3):e0265677. doi: 10.1371/journal.pone.0265677 (PMC8970403; doi:10.1371/journal.pone.0265677)
Supplement: Attachment — Submitted filename: Answers to the Reviwers Comments.docx [file pone.0265677.s003.docx]

Reviewers' comments:

Reviewer's Responses to Questions

**Comments to the Author**

1. Is the manuscript technically sound, and do the data support the conclusions?

The manuscript must describe a technically sound piece of scientific research with data that supports the conclusions. Experiments must have been conducted rigorously, with appropriate controls, replication, and sample sizes. The conclusions must be drawn appropriately based on the data presented.

Reviewer #1: Yes

Reviewer #2: No

2. Has the statistical analysis been performed appropriately and rigorously?

Reviewer #1: Yes

Reviewer #2: No

3. Have the authors made all data underlying the findings in their manuscript fully available?

The [PLOS Data policy](http://www.plosone.org/static/policies.action#sharing) requires authors to make all data underlying the findings described in their manuscript fully available without restriction, with rare exception (please refer to the Data Availability Statement in the manuscript PDF file). The data should be provided as part of the manuscript or its supporting information, or deposited to a public repository. For example, in addition to summary statistics, the data points behind means, medians and variance measures should be available. If there are restrictions on publicly sharing data—e.g. participant privacy or use of data from a third party—those must be specified.

Reviewer #1: Yes

Reviewer #2: Yes

4. Is the manuscript presented in an intelligible fashion and written in standard English?

PLOS ONE does not copyedit accepted manuscripts, so the language in submitted articles must be clear, correct, and unambiguous. Any typographical or grammatical errors should be corrected at revision, so please note any specific errors here.

Reviewer #1: Yes

Reviewer #2: No

5. Review Comments to the Author

Please use the space provided to explain your answers to the questions above. You may also include additional comments for the author, including concerns about dual publication, research ethics, or publication ethics. (Please upload your review as an attachment if it exceeds 20,000 characters)

Reviewer #1: 1. Paper title: “High-Strength Concrete Beams” should be “Reinforced High-Strength Concrete Beams”. Otherwise, it may confuse readers that the discussed objects are plain concrete beams.
2. Line 24: “to 0.1” should be revised to be “as 0.1”.
3. Introduction part: there are too many small paragraphs which only contain one or two sentences in this section. Please carefully revise the structure of this section.
4. Section 2: In this section, the authors reviewed many classical literature and main findings. However, they did not introduce any formulas proposed by existing scholars. Since the predictive formulas is the research topic of this paper (see the last paragraph of section 2), the reviewer thinks more information should be added in this part.
5. Lines 205~209:In this section, the authors mentioned that they collected many experimental records from available studies. However, they did not give any filtering criteria about the database. This will probably make the database untrustworthy. The reviewer thinks the authors should add some messages (May see paper “Machine Learning-Based Evaluation of Shear Capacity of Recycled Aggregate Concrete Beams” for reference).
6. Please mark the coefficient of correlation directly in figures 1~3, this can be convenient for readers to see the predictive results.
7. Table 4: The authors listed a series of predictive expressions in this table. However, they forgot to inroduce the application ranges for these formulas, and also did not check the availability of the collected database to each present expression (at least did mention it in the paper). So please carefully do this aspect of works.
8. Conclusions: Please carefully revise the Bullets 1, 3, 4 and 6 in this section. The reviewer thinks that some of them are not innovative and clear, and some of them are not found by the present study.

Reviewer #2: This paper presents a data analysis for the reported results for shear strength of HSC beams. Different predicting models are proposed based on regression analysis. In general, the paper is of poor quality and the methodology adopted by the author is not justified. Detailed comments are as follows.
1. Please make the title shorter.
2. Literature review simply lists the works done by other researchers without critical review, summary and discussion. The novelty of the present study is not clear.
3. The shear strength models are simply proposed based on the regression of collected data from literature, and the data classification only depends on whether steel fibre is included or not, however, many different factors would influence the shear behaviour of a concrete beam. The method used by the author would lead to unreliable prediction.
4. The paper has to be edited by a native English speaker.

6. PLOS authors have the option to publish the peer review history of their article ([what does this mean?](https://journals.plos.org/plosone/s/editorial-and-peer-review-process#loc-peer-review-history)). If published, this will include your full peer review and any attached files.


If you choose “no”, your identity will remain anonymous but your review may still be made public.


**Do you want your identity to be public for this peer review?** For information about this choice, including consent withdrawal, please see our [Privacy Policy](https://www.plos.org/privacy-policy).

Reviewer #1: No

Reviewer #2: No

**Reviewers' comments:**

Reviewer's Responses to Questions

**Comments to the Author**

1. Is the manuscript technically sound, and do the data support the conclusions?

The manuscript must describe a technically sound piece of scientific research with data that supports the conclusions. Experiments must have been conducted rigorously, with appropriate controls, replication, and sample sizes. The conclusions must be drawn appropriately based on the data presented.

Reviewer #1: Yes

Reviewer #2: No

**Answer: 1**

This study depends on the experimental data found in literatures which are done carefully and conducted rigorously and published in known and original scientific journals. The conclusions are drawn on the results of the proposed equations and comparison of (26) available mathematical models. Twelve proposed mathematical equations are proposed to predict shear strength of reinforced high strength concrete beams with and without steel fiber taking the effect of concrete compressive strength $(f_{c}^{'})$, flexural longitudinal reinforcement index $(\rho_{l})$ and shear span ratio $(\frac{a}{d})$ into account, in addition of the steel fiber properties/volumetric volume content $Q_{f}\%$, aspect ratio $(\frac{L}{d})$ and fiber type which is represented by bond factor).

2. Has the statistical analysis been performed appropriately and rigorously?

Reviewer #1: Yes

Reviewer #2: No

**Answer:2**

The statistical analysis are performed appropriately and rigorously in the following steps.

a. Derivation of set of simultaneous equations in matrix form based on the principle of least squares method for all proposed mathematical models
b. The values of the matrix coefficients are determined in corperation with the (245) experimental tests of reinforced concrete beams found in literatures, and using Excel data sheet.
c. Solution of the simultaneous equations using Matlab software to find the values of the constants of the proposed model.
d. Return to the same data sheet, the theoretical results of the shear strength are determined by application of the proposed mathematical model as the parameter of the study ($f_{c}^{'}, \rho_{l}, \frac{a}{d},$and steel fiber properties).
e. The theoretical results are compared with the experimental and statistical parameters are determined such as (Ratio of experimental shear to theoretical shear strength $(\frac{V_{c test}}{V_{c cal}})$, standard deviation $(\sigma)$, variance (var.) and coefficient of correlation (r).
f. Finally, the plot of the experimental shear versus predicted shear is drawn to show validity of the proposed model.
g. These procedures are repeated for all proposed models.

3. Have the authors made all data underlying the findings in their manuscript fully available?

The [PLOS Data policy](http://www.plosone.org/static/policies.action#sharing) requires authors to make all data underlying the findings described in their manuscript fully available without restriction, with rare exception (please refer to the Data Availability Statement in the manuscript PDF file). The data should be provided as part of the manuscript or its supporting information, or deposited to a public repository. For example, in addition to summary statistics, the data points behind means, medians and variance measures should be available. If there are restrictions on publicly sharing data—e.g. participant privacy or use of data from a third party—those must be specified.

Reviewer #1: Yes

Reviewer #2: Yes

**Answer:3**

Yes

4. Is the manuscript presented in an intelligible fashion and written in standard English?

PLOS ONE does not copyedit accepted manuscripts, so the language in submitted articles must be clear, correct, and unambiguous. Any typographical or grammatical errors should be corrected at revision, so please note any specific errors here.

Reviewer #1: Yes

Reviewer #2: No

**Answer:4**

Done

5. Review Comments to the Author

Please use the space provided to explain your answers to the questions above. You may also include additional comments for the author, including concerns about dual publication, research ethics, or publication ethics. (Please upload your review as an attachment if it exceeds 20,000 characters)

Reviewer #1: 1. Paper title: “High-Strength Concrete Beams” should be “Reinforced High-Strength Concrete Beams”. Otherwise, it may confuse readers that the discussed objects are plain concrete beams.
2. Line 24: “to 0.1” should be revised to be “as 0.1”.
3. Introduction part: there are too many small paragraphs which only contain one or two sentences in this section. Please carefully revise the structure of this section.
4. Section 2: In this section, the authors reviewed many classical literature and main findings. However, they did not introduce any formulas proposed by existing scholars. Since the predictive formulas is the research topic of this paper (see the last paragraph of section 2), the reviewer thinks more information should be added in this part.
5. Lines 205~209:In this section, the authors mentioned that they collected many experimental records from available studies. However, they did not give any filtering criteria about the database. This will probably make the database untrustworthy. The reviewer thinks the authors should add some messages (May see paper “Machine Learning-Based Evaluation of Shear Capacity of Recycled Aggregate Concrete Beams” for reference).
6. Please mark the coefficient of correlation directly in figures 1~3, this can be convenient for readers to see the predictive results.
7. Table 4: The authors listed a series of predictive expressions in this table. However, they forgot to inroduce the application ranges for these formulas, and also did not check the availability of the collected database to each present expression (at least did mention it in the paper). So please carefully do this aspect of works.
8. Conclusions: Please carefully revise the Bullets 1, 3, 4 and 6 in this section. The reviewer thinks that some of them are not innovative and clear, and some of them are not found by the present study.

**Answer:5**

Reviewer #1:

1. The title should be changed to “Reinforced High-Strength Concrete beams…” Done
2. Done as requested
3. Done as requested
4. The Literature Review has been updated with current day.
5. Large database of experimental rests are found in literature, then the data is filtered to choose the data which fit with the purpose of this study, which is the prediction of shear strength of the reinforced high strength concrete beams with and without steel fibers, so the data is already filtered, then used in the analysis procedures.
6. Done as requested
7. Table 4: The range of the experimental data and range of application of the mathematical proposed equations are:

Concrete compression strength $(f_{c}^{'})$ = 20-90 mpa

Flexural longitudinal reinforcement index $(\rho_{l}\%)$ =1.3 - 3.3%

Shear span ratio $(\frac{a}{d})$ = 2-5	Done

8. Revise the conclusions 1,3,4 & 6

1- The shear strength of reinforced high strength concrete beams are determined in term of variables taken into account, such as concrete compressive strength $\left( f_{c}^{'} \right)$, flexural longitudinal reinforcement index $(\rho_{l})$, and shear span ratio $(\frac{a}{d})$.

3- Because of complicated behavior of the shear strength, more researches are necessary in the field of reinforced high strength beams with and without steel fibers.

4- Different mathematical models are proposed based on the principle of least squares using different methods of regression analysis (linear, multilinear, and nonlinear).

6- The theoretical results of the proposed equations are very close and shared excellent correlation to the experimental data.

Reviewer #2: This paper presents a data analysis for the reported results for shear strength of HSC beams. Different predicting models are proposed based on regression analysis. In general, the paper is of poor quality and the methodology adopted by the author is not justified. Detailed comments are as follows.
1. Please make the title shorter.
2. Literature review simply lists the works done by other researchers without critical review, summary and discussion. The novelty of the present study is not clear.
3. The shear strength models are simply proposed based on the regression of collected data from literature, and the data classification only depends on whether steel fibre is included or not, however, many different factors would influence the shear behaviour of a concrete beam. The method used by the author would lead to unreliable prediction.
4. The paper has to be edited by a native English speaker.

**Answer:5**

Reviewer #2:

1. Shorter title: “write new name” Done
2. The novelty of this study is proposing different mathematical models to predict shear strength of reinforced high strength concrete beams including the effect of steel fiber properties (volumetric volume content, aspect ratio, and fiber type) in addition to concrete compressive strength, flexural longitudinal reinforcement index and shear span ratio.

The main novelty of this study is focusing on

a. Study of shear strength of reinforced high strength concrete beams.
b. Including the effect of steel fiber properties.
c. The relation of the shear strength with the concrete compressive strength (especially with the power of $f_{c}^{'}$).
3. The main purpose of this study is determining the real relationship and power of concrete compressive strength with the shear strength of concrete beams with wide range of ($f_{c}^{'}$ = 20-90 mpa), also including the effect of the steel fiber properties which is represented by Fiber factor (F). The theoretical results obtained from the proposed equations five excellent correlation and very close to the experimental data for both concrete beams with and without steel fiber.
4. Done
