# Supplementary material for: Prediction and developing of shear strength of reinforced high strength concrete beams with and without steel fibers using multiple mathematical models
Source: PLoS One. 2022 Mar 31;17(3):e0265677. doi: 10.1371/journal.pone.0265677 (PMC8970403; doi:10.1371/journal.pone.0265677)
Supplement: Attachment — Submitted filename: Answers to reviewers comments.docx [file pone.0265677.s004.docx]

Review Comments to the Author

**Reviewer #1**

The authors have clearly answered my questions. In the reviewer's opinion, this manuscript can be accepted in the current form.

The following two papers are provided for the authors for reference:

[1] Yong Yu, Xinyu Zhao, Jinjun Xu, Cheng Chen, Simret Tesfaye Deresa, Jintuan Zhang. Machine learning‐based evaluation of shear capacity of recycled aggregate concrete beams. Materials, 2020, 13(4552).
[2] Xie T. Y., Aliakbar G., Togay O. Toward the development of sustainable concretes with recycled concrete aggregates: Comprehensive review of studies on mechanical properties. Journal of Materials in Civil Engineering, 2018, 30(9), 04018211.

| Reviewer Comment | 1 |
| --- | --- |
| Authors’ response | First of all, the author appreciates the overall evaluation of the paper, as well as thorough the valuable suggestions.  Based on the reviewer’s remark, the mentioned papers have been added to the relevant part of the manuscript. In addition, two new paragraphs were added accordingly. |
| Notes/action | Please, see the highlighted paragraphs in section 2 (Yalow color)  Existing major shear design formulae established primarily for conventional concrete beams were assessed for recycled aggregate concrete (RAC) beams. Results showed that when applied to the shear test database compiled for RAC beams, those formulae provided only inaccurate estimations with surprisingly large scatter. To cope with this bias, machine learning (ML) techniques deemed as potential alternative predictors were resorted to. First, a Grey Relational Analysis (GRA) was carried out to rank the importance of the parameters that would affect the shear capacity of RAC beams. Then, two contemporary ML approaches, namely, the artificial neural network (ANN) and the random forest (RF). It was found that both models produced even better predictions than the evaluated formulae. With this superiority, a parametric study was undertaken to observe the trends of how the parameters played roles in influencing the shear resistance of RAC beams. The findings indicated that, though less influential than the structural parameters such as shear span ratio, the effect of the replacement ratio of recycled aggregate (RA) was still significant, for safe application of RAC, using partial factors calibrated to consider the uncertainty is feasible when designing the shear strength of RAC beams [25].  The use of recycled concrete aggregates as an alternative aggregate material in concrete, where natural aggregates are replaced with recycled concrete aggregates, is a promising technology for conserving natural resources and reducing the environmental impact of concrete. Xie et al [26] presents a study on mechanical and durability properties of concretes manufactured with recycled aggregates of different sizes and contents. A total of 14 batches of RACs were manufactured. The compressive strength, elastic modulus, flexural strength, splitting tensile strength, workability, drying shrinkage, and water absorption of each batch are experimentally tested. Test following parameters are considered (recycled aggregate replacement ratio, size of coarse aggregates, and mixing method used in the preparation of concrete). The results indicate that the compressive strength is an important factor on mechanical and durability-related properties of RACs in addition to the properties of different RAC mixes of the same compressive strength such as (the size and content of the coarse aggregates).  New References:   25 Yong Yu, Xinyu Zhao, Jinjun Xu, Cheng Chen, Simret Tesfaye Deresa, Jintuan Zhang. Machine learning‐based evaluation of shear capacity of recycled aggregate concrete beams. Materials, 2020. 26 Xie T. Y., Aliakbar G., Togay O. Toward the development of sustainable concretes with recycled concrete aggregates: Comprehensive review of studies on mechanical properties. Journal of Materials in Civil Engineering, 2018, 30(9), 04018211. |

**Reviewer #2**

1. In Section 2, the author lists many research results, but only makes a brief statement and summary of each researcher's conclusions without any critical review or analysis of their progress or limitations. Reviewers believe that the Section 2 should have more discussion rather than a simple statement.

| Reviewer Comment | 1 |
| --- | --- |
| Authors’ response | First of all, the authors appreciate the reviewer’s comprehensive comments, which further improved the quality of the paper.  Indeed, there was not detailed discussion and critical review regarding the selected papers. In order to be easily read by the readers, the discussions and further details were added to some of the models that they missed relevant parameters, At the end, as recommended by the reviewer, a critical review were made.  **RELEVANT PARAMETERS:**  See the paragraphs below  **CRITICAL REVEW:**  See the paragraph below |
| Notes/action | Please, see the highlighted paragraphs in section 2 (Green color)  **RELEVANT PARAMETERS:**  Line 75-78:  They cast and tested ten reinforced concrete beams with dimensions (width b=100 mm, effective depth d=180 mm), shear span ratio constant for all beams (a/d=2.833) and the concrete compressive strength (fc’) ranges (21 to 74 MPa), all beams reinforced with same reinforcement ratio (ρ=0.0131). They concluded that the repaired beams showed a lower stiffness and greater ductility than the original beams.  Line 81-86:  Tahenni et al. [10] presented an experimental work on the shear behavior of HSC beams with steel fiber. They cast and tested eight reinforced concrete beams with steel fibers content (0, 0.5, 1 and 2% by volume) and two aspect ratios (65 and 80). The beam dimensions (width b=100 mm, effective depth d=136 mm), shear span ratio constant for all beams (a/d=2.2) and the concrete compressive strength (fc’) is the same for all beams (65 MPa), all beams reinforced with the same reinforcement ratio (ρ=0.0116).  Line 97-102:  Radmila [2] casted and tested 26 normal and high strength concrete reinforced concrete beams with (b=120 mm) and different values of effective depth d=180, 240, 300 and 360 mm), shear span ratio (a/d=1.25, 1.5, 2 and 2.67) and the concrete compressive strength (fc’=35 MPa) for normal concrete and (fc’=90 MPa) for high strength concrete, reinforcement ratio ranges (ρ=0.015 to 0.033). proposed a new equation for prediction of shear strength of HSC beams, based on the experimental researches.  Line 123-129:  Reddy et al. [6] presented some improvement on the prediction equation of shear strength of fibrous HSC beams with and without web reinforcement. They cast and tested 16 reinforced concrete beams with dimensions (width b=100 mm, effective depth d=130 mm), the concrete compressive strength (fc’= 70 MPa) for all beams, and the same reinforcement ratio is used (ρ=0.0725). The beams with different shear span ratios $(\frac{a}{d}=1,2,3, and 4)$ and various volumetric fiber content (0.4, 0.8 & 1.2%) and aspect ratio (75). The test results indicated an increase in the cracking shear resistance and ultimate shear strength.  Line 155-159:  Saghair et al. [16] presented an experimental study on shear behavior of HSC beams by testing 19 beams with dimensions (width b=120 mm and effective depth d=275 mm), shear span ratio (a/d=1, 2, 3 and 4) are used, concrete compressive strength (fc’=50 and 80 MPa) for high strength concrete and (30 MPa) for normal concrete beams and the reinforcement ratio (ρ=0.00515) for all beams.  Line 165-167:  Mphonde & Frantz [17] presented an experimental study by testing 12 beams with a constant shear-span ratio $\frac{a}{d}=3.6$ and concrete compression strength vary from 21 to 84 MPa. The beamwidth b=150 mm, effective depth d=298.5 mm and longitudinal reinforcement ratio (0.0329).  Line 171-173:  Fujita et al. [18]; presented an experimental investigation on the shear strength of high-strength reinforced concrete beams with concrete compressive strength ranging 36-100 MPa and the shear-span ratio of 2-5, different beam dimensions (b , d) are used (150, 250), (150, 500) and (350, 1000) mm and longitudinal reinforcement ratio ranges (0.0153 to 0.0136).  Line 178-180:  Kolhapure [19] presented an experimental investigation on twelve HSC beams of dimensions (b=125 mm and d=100 mm) and concrete compressive strength (65 MPa), including the effect of shear-span ratio (a/d=3 and 5), longitudinal reinforcement ratio ranges (0.008 to 0.032) and minimum web reinforcement.  Line 200-204:  Ashour et al [23] tested 18 rectangular high-strength fiber reinforced concrete beams with dimensions (b=125 mm and d=215 mm), the beams are subjected to combined flexure and shear. The main variables were the steel fiber content (0.5 and 1%) by volume and aspect ratio (75), the longitudinal steel reinforcement ratio ranges (0.0284 to 0.0458) and the shear-span ratio (1, 2, 5 and 6), the concrete compressive strength ranges (92 to 101 MPa).  **CRITICAL REVEW:**  Lines 243-255 (NEW paragraph)  Based on the results given in the literature [3, 12, 29-52], it can be said that most of the models were underestimated the average ratio of $(R_{avg})$ that have been found based on the experimental to predicted shear $(R_{avg}>1.0)$. For example, the models are given by the ACI-Code [29, 31], Kim and Wight [32], CEP-FIP 1990 [36] and 1993 [37], CSA94 [38], EHE 99 [41], EC2002 model [42], AS3600 model [43], IS456:2000 model [45], Arsalan [47], JSCE 1996 [49], and Bazant & Kim model [50], where the ${(R}_{avg})$ ranged between (1.142 and 2). While the results of other models are overestimated, the average ratio ${(R}_{avg}<1.0)$. For example, the models are proposed by Hammad et al. model [3], Zsulty model [39], NZS3101 model [44], Niwa et al model [51], Bazant & Kim model [52], the ${(R}_{avg})$ value ranged between (0.637 and 0.922). Nevertheless, the following models showed acceptable results of shear strength such as ACI-Code model [30], Russo et al model [33], Bae et al model [12], CEP-FIP model [35] and Gastebled & May model [40], where the ${(R}_{avg})$ value near the unity but they are still not close to one. |

2. In Section 4, the author has collected a large amount of data, what criteria are used to select these data? please give more information and explanation to ensure the authenticity and reliability of these data.

| Reviewer Comment | 2 |
| --- | --- |
| Authors’ response | Once again, thank you very much for your valuable comment. Indeed, this part has been missed in the manuscript.  Based on the reviewer’s remark, the criteria was added to the manuscript.  Please, see the last paragraph in section 4. |
| Notes/action | Please, see the last paragraph (line 283-293) in section 4. (Green color)  Additionally, different types of models are proposed including all possible combinations of the variables mentioned in Section 3, then the best model is recommended to predict shear strength of HSC beams with and without steel fiber, also the results are compared with different equations found in available codes and recommendations. In order to ensure the authenticity and reliability of these data, four strict criteria were selected, i.e. (i) the main focus was made on the data given by international standards and codes (practical guidance), then scientific papers; (ii) the selected study must be published in the reliable journal, especially Scopas and Clarivate; (iii) The selected data must be related to the high strength concrete; (iv) All selected studies must have enough data to find the coefficient of correlation (r), standard deviation (σ), variation, R_average_, R_maximum_ and R_minimum_. In addition, the experimental results of previous studies showed that the shear strength of concrete beams $(v_{c})$ depends on the concrete compressive strength $(f_{c}^{'})$, longitudinal reinforcement index $(\rho)$, and shear span ratio $(\frac{a}{d})$. The available models of predicting $(v_{c})$ from the codes and references also depend on these parameters, that is why these parameters are selected from the literatures and previous studies to propose new models for predicting shear strength of high strength concrete beams. |

3. Some figures do not highlight the advantages of the model obtained in this study over other models. It is suggested that the author make appropriate modifications to these figures (such as Fig.10). In addition, there are many statements irrelevant to the research of this paper in the conclusion part. Please make appropriate modifications to highlight the innovations of this research.

| Reviewer Comment | 3 (A and C) |
| --- | --- |
| Authors’ response | Once again, thank you very much for another valuable remark. The authors agree with the reviewer’s remark. For example, the old figure was not shown the advantage of the proposed model. Thus, A NEW FIGURE was drawn to clarify the output of this study. Please, check Fig. 10 in the new manuscript (also shown below).  Also, in lines 390-395, further details regarding the proposed model has been shown in the new manuscript, namely the novelty or the output of this work. |
| Notes/action | Please, see lines 390-395 in section 6 (Green color)  The multilinear and nonlinear regression analysis are used to propose different models for predicting shear strength $(V_{c})$ of HSC concrete beams, in terms of the main variables mentioned above. The predicted results from the proposed equations are compared with (26) available equations found in previous studies, codes, and standards. As shown in Figure 10, equations [29- 32, 36-38, 41-43, 45, 47, 49-50] underestimated and equations [3, 39, 44, 51-52] overestimated the shear strength. However, some equations [12, 30, 33, 35, 40] seem that they relatively have a good compatible unity but their R_ave_ are still not close to one as well as their variation value is very high. While, the proposed models (EP), namely PE3 (Equation 8) and PE4 (Equation 9) are very compatible to unity with low variation value.  NEW FIGURE:  Please, check the figure below |

Figure 10:

|  | Ravg (Vc exp/Vc cal) | Var. |
| --- | --- | --- |
| ACI-Code equation | 1.179 | 0.296 |
| ACI-Code equation | 1.096 | 0.256 |
| ACI-Code equation | 1.21 | 0.313 |
| Kim & Wight: | 1.652 | 0.67 |
| Russo et al. | 1.049 | 0.222 |
| Bazant & Kim | 0.765 | 0.138 |
| Bae et al | 0.949 | 0.328 |
| CEP-FIP | 1.064 | 0.189 |
| CEP-FIP 1990 | 1.33 | 0.295 |
| CEP-FIP 1993 | 1.404 | 0.343 |
| CSA 94 | 1.119 | 0.198 |
| Zsutty | 0.831 | 0.19 |
| Hammad et al | 0.637 | 0.097 |
| Gastebled & May | 1.048 | 0.141 |
| EHE 99 Spanish | 2 | 1.471 |
| EC 2002 | 1.4 | 0.342 |
| AS3600 | 1.35 | 0.344 |
| NZS 3101 | 0.778 | 0.208 |
| IS456:2000 | 1.635 | 0.628 |
| BS8101 | 0.922 | 0.12 |
| Arsalan 2008 | 1.258 | 0.342 |
| Russo et al 2013 | 0.967 | 0.162 |
| JSCE 1996 | 1.142 | 0.219 |
| Bazant & Kim | 1.341 | 0.348 |
| Niwa et al | 0.872 | 0.127 |
| Bazant & Kim | 0.782 | 0.145 |
| PE1 (Eq.6) | 1.09 | 0.273 |
| PE2 (Eq.7) | 1.095 | 0.3 |
| PE3 (Eq.8) | 1 | 0.224 |
| PE4 (Eq.9) | 0.984 | 0.279 |
| PE5 (Eq.11) | 1.1875 | 0.324 |
| PE6 (Eq.12) | 1.185 | 0.327 |

| Reviewer Comment | 3 (\|B) |
| --- | --- |
| Authors’ response | Regarding the conclusion section, A major correction was considered based on the reviewer’s remark. |
| Notes/action | Please, see section 7 (Green color)  To predict shear strength (V_c_) of HSC beam with and without steel fibers, a large database of experimental results is collected in previous studies and applied on 26 available equations found in previous studies, codes, and standards.  The shear strength of HSC beams is proportional to the concrete compressive strength of power about 0.1, while the most available equation in codes and standards, the shear strength is proportional to $(f_{c}^{'})$ of the power (0.5).  The results show that most of the proposed models predict the shear strength of the HSC beam in away either it is overestimated or underestimated. Nevertheless, few models relatively have a good compatible unity but their R_ave_ ($\frac{V_{excpermental}}{V_{calculated}}$) are still not close to one. Additionally, their variation value is very high. Thus, the multilinear and nonlinear regression analysis are used to propose different models for predicting $V_{c}$ of HSC concrete beam accurately. The proposed models are very compatible to unity with low variation value. In addition, the new models proposed by this study are including the fiber factor (F) which represents the fiber properties (fiber content $Q_{f}\%$, fiber aspect ratio $\frac{L}{d}$, and fiber type) in the equation of shear strength in addition to other variables considered in this study. Apart from the proposed models, the shear behavior of HSC is complicated and not well-understood. That is why more research is necessary to study its performance in shear resistance and to find the best equation to predict it. |

Finally:

Based on the reviewer’s remark, the mentioned papers have been added to the relevant part of the manuscript. In addition, two new paragraphs were added accordingly.
